# A description of the gross pathology of drowning and other causes of mortality in seabirds

**DOI:** 10.1186/s12917-017-1214-1

**Published:** 2017-10-12

**Authors:** Victor R. Simpson, David N. Fisher

**Affiliations:** 1 Wildlife Veterinary Investigation Centre, Chacewater, Truro, Cornwall TR4 8PB UK; 2 Centre for Ecology and Conservation, University of Exeter, Penryn, Cornwall TR10 9FE UK; 3 Present Address: Department for Integrative Biology, University of Guelph, Guelph, ON N1G 2W1 Canada

**Keywords:** Seabird, Auk, Stranding, Drowning, Bycatch, Pathology, Air sac, Oil, Polyisobutylene

## Abstract

**Background:**

Mortality of seabirds due to anthropogenic causes, especially entrapment in fishing gear, is a matter of increasing international concern. This study aimed at characterising the gross pathology of seabirds that drowned in fishing nets and comparing it with that in other common causes of mortality.

**Results:**

Post-mortem examinations were performed on 103 common guillemots, 32 razorbills, 37 shags and 5 great northern divers found stranded in Cornwall during 1981–2016. Pathology in birds that died in confirmed incidents of drowning in fishing nets (*n* = 95) was compared with that in cases of suspected drowning (*n* = 6), oil (*n* = 53) and polyisobutylene (PIB) (*n* = 3) pollution, adverse weather (n = 6) and stranding of unknown cause (14). The majority of drowned birds were in good nutritional state, freshly dead and approximately 50% had freshly ingested fish in their proximal gut. Principle lesions were: gross distention of the heart and major veins with dark blood, intensely congested, swollen and oedematous lungs which released white frothy fluid when excised, watery fluid in the air sacs that ranged from clear to deep red depending on state of carcase preservation. PIB-affected birds were in good nutritional state; their pathology was largely consistent with that in confirmed drowning cases; it is likely that drowning was the ultimate cause of death. Birds affected by oil, adverse weather or that stranded due to unknown cause were all in poor or emaciated condition, the mean body mass of the guillemots and razorbills being, respectively, 53 and 57% of those that drowned. They had little or no food in their alimentary tracts and many showed evidence of enteric inflammation, haemorrhage and ulceration. None had fluid in their air sacs and none showed significant cardio-respiratory system lesions.

**Conclusions:**

Drowned birds consistently showed a distinctive set of gross pathological lesions. When combined with contemporaneous observations, the pathology may be sufficient to permit a diagnosis of drowning, especially where a batch of freshly dead birds are examined. The observations in this study are likely to be of value when investigating stranding incidents, particularly where it is suspected that legislation aimed at protecting seabirds is not being complied with.

**Electronic supplementary material:**

The online version of this article (10.1186/s12917-017-1214-1) contains supplementary material, which is available to authorized users.

## Background

In recent decades there has been increasing international concern over the level of seabird mortality caused by human activities, the most significant of which are entrapment in fishing gear (bycatch) and oil pollution. Global estimates of mortality caused by the fishing industry are imprecise but it is suggested that 400,000 birds die annually in gill nets and 320,000 in longline fisheries [1]. In the North Sea and Baltic it is believed that at least 90,000 waterbirds drown in nets annually and that the true number could be as high as 200,000 [2]. Major oil spill incidents, which often affect tens or even hundreds of thousands of birds, are well publicised. However, innumerable small oil spills go largely unrecorded but are believed to be responsible for greater mortality. In Newfoundland alone the mortality from repeated low level inputs of oil, so-called chronic pollution, is estimated to average 315,000 birds annually [3]. Chronic oil pollution is also a major concern in Europe, especially in the North West European waters [4]; these include the English Channel and the Celtic sea.

Cornwall is a maritime county in south-west England where commercial fishing, shipping and tourism are major contributors to the local economy. It is also rich in marine wildlife and the relatively natural 700 Km coastline, bounded by the English Channel to the south and the Celtic sea to the north, supports large populations of seabirds. Incidents of stranded dead or dying birds are common and post-mortem examinations frequently identify anthropogenic factors as the cause. The purpose of this report is to characterise the gross pathology seen in common guillemots (*Uria aalge* **)**, razorbills (*Alca torda*), shags (*Phalacrocorax aristotelis*) and great northern (GN) divers (*Gavia immer*) that had drowned and to compare this with the pathology seen in birds that died due to oil pollution or other causes.

## Methods

This was an opportunistic study of seabirds submitted for post-mortem examination by the Royal Society for the Prevention of Cruelty to Animals (RSPCA), various conservation bodies, government agencies and members of the public. The birds had either been found dead, moribund or sick along the coast of Cornwall. Dead and moribund birds were sent directly for post-mortem examination whilst sick birds, many of which showed clear evidence of oil pollution, were treated at a wildlife hospital; those that died or were euthanased due to a poor prognosis were normally submitted for post-mortem examination within 24 h. In incidents involving large numbers of birds a representative sample, usually of ten to fifteen birds, was examined. Between 1981 and 2001 the post-mortems were performed at the Veterinary Laboratories Agency (VLA), Truro, and thereafter, until 2016, at the Wildlife Veterinary Investigation Centre. In most incidents the birds were examined within 24 h of them being found but in a few cases carcases were either submitted frozen or a proportion were frozen on receipt for logistical reasons.

Each bird was given a unique reference number and examined following a standard protocol [5]. The body mass, excluding ingesta mass, was recorded in all cases. Liver mass was recorded in most cases and pectoralis muscle mass in drowning cases; however, in some incidents constraints on time prevented all these parameters from being recorded. Age class was determined as sub-adult or adult by the degree of gonad development. In selected cases parasites were submitted to Dr. E. Harris at the Natural History Museum, London for identification. Samples of watery fluid were taken from the air sacs of selected drowned birds and examined by direct light microscopy for the presence of plankton, diatoms or other marine contaminants.

Statistical analyses were performed in the software R ver. 3.1.2 [6]. Only birds with a known cause of death were included in the analyses. To determine whether birds differed in body mass due to cause of death, sex and species, a linear model (LM) was used with the log of body mass as a response (this was necessary to achieve normality), and age (adult or sub-adult), sex, species and cause of death (either drowned or non-drowned) as predictor variables. The LM also included a sex-species interaction, a species-age interaction, and an age-cause of death interaction, to account for any differences between the species in sexual dimorphism, variation in the difference between age classes between the species, and variation in the difference between drowned and non-drowned masses between age classes respectively.

Linear models were also used to analyse organ mass relative to body mass, as it might be expected that when a bird loses condition its organs may not regress in the same way. Therefore, for each of liver and pectoral muscle mass, LMs were used with organ mass set as a response and body mass, sex, age, and species as predictor variables. The cause of death was included as a predictor in the model for liver mass but could not be included in the model for pectoral muscle mass as this was only recorded in those birds that had drowned. The model for liver mass included an interaction between mass and cause of death (to determine whether birds that died in a certain way had different body mass-liver mass relationships). These LMs used a normal error structure.

In all cases, terms were removed and resulting models tested for significant differences with the previous model using F tests. The first terms to be removed were those with the highest *p*-values. Single terms were not tested until any interactions involving them had been removed. If species, or an interaction involving it, was found to be significant, we conducted pair-wise comparisons for each species pair by removing the other species from the dataset and re-analysing the subset with the final model. This allowed us to directly compare one species against another, while controlling for other factors e.g. cause of death or body mass (for organ mass).

## Results

Post-mortem examinations were performed on 177 birds. More than half of these (*n* = 95) came from eight observed drowning incidents. The rest were from four suspected but unconfirmed drowning incidents, 15 oil pollution incidents, a single confirmed incident of polyisobutylene (PIB) pollution and a suspected ‘storm wreck’ (mass death of debilitated birds due to extreme weather conditions). There were six incidents where birds became stranded for no obvious reason and where their gross appearance, and the circumstances, such as location and weather conditions, did not suggest a likely cause. This category of mortality is hereafter referred to as unknown cause. The species and number of birds examined in each type of incident are summarised in Table 1.

**Table 1:** The number of mortality incidents by category of death and the species and numbers of birds examined in each category

Incident type & number^.^	Number & species of birds examined	Total
Drowning, confirmed *n* = 8	39 guillemots, 25 razorbills, 26 shags, 5 GN divers	95
Drowning, unconfirmed, 4	1 guillemot, 3 razorbills, 2 shags	6
PIB pollution, *n* = 1	3 guillemots	3
Oil pollution, *n* = 15	48 guillemots, 3 razorbills, 2 shags	53
Unknown cause, *n* = 6	12 guillemots, 1 razorbill, 1 shag	14
Storm wreck, n = 1	6 shags	6

## Gross pathology in each category of death

### Confirmed drowning

#### External features

Five of the eight observed incidents of drowning in fishing nets involved guillemots or razorbills, either together or as a single species. The birds ranged from freshly dead to slightly autolysed. Their plumage was usually wet but in good condition and a few birds had slight scavenger damage. Body condition in the great majority of cases was good with well-developed muscles and good to excellent subcutaneous fat deposits. A sixth confirmed incident involved 26 shags, two guillemots and a razorbill. Their plumage was in good condition, although very wet and contaminated by sand; two shags showed signs of scavenger damage and some birds showed evidence of mild autolysis. Two of the shags and a guillemot were examined within 24 h of submission and the rest after being stored at minus 20^o^ C. The majority of the shags and the razorbill were in good muscular condition with good to excellent subcutaneous fat deposits but the guillemots were only in poor to fair condition. Thirteen of the shags and one guillemot had localised dark red, irregular, subcutaneous areas of suspected bruising, often with associated oedema. These lesions were mostly around the neck but also over the shoulders, sternum or the lateral aspect of the thighs. Large numbers of subcutaneous mites (*Laminosioptes* sp.) were present in the shags, particularly in the precrural area. Five GN divers died in the other two confirmed incidents. All were submitted freshly dead and the plumage in each case was in good condition. One diver had monofilament nylon net enclosing the left carpal joint and another had net around the joints of both wings which prevented them from being extended; the right hock joint of the latter bird was dislocated. Muscle condition was good in all five birds and subcutaneous fat deposits ranged from fair to excellent. Three divers were examined in fresh condition and two after being stored at minus 20^o^ C.

#### Internal features

**Fig. 1:**
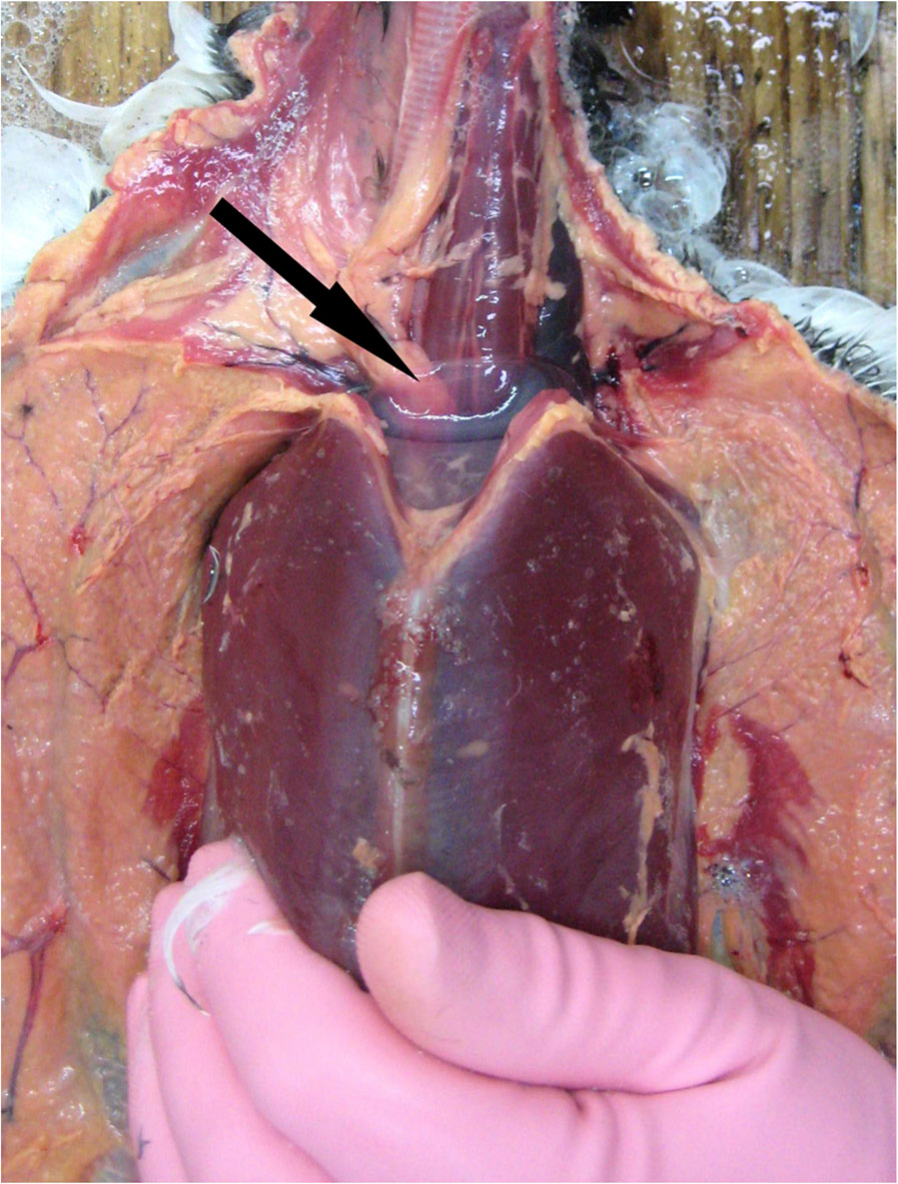
Visualisation of watery fluid in the clavicular air sac. When fluctuating downward pressure was applied to the abdomen of this drowned guillemot clear watery fluid was readily observed moving in the distended clavicular air sac (arrow)

**Fig. 2:**
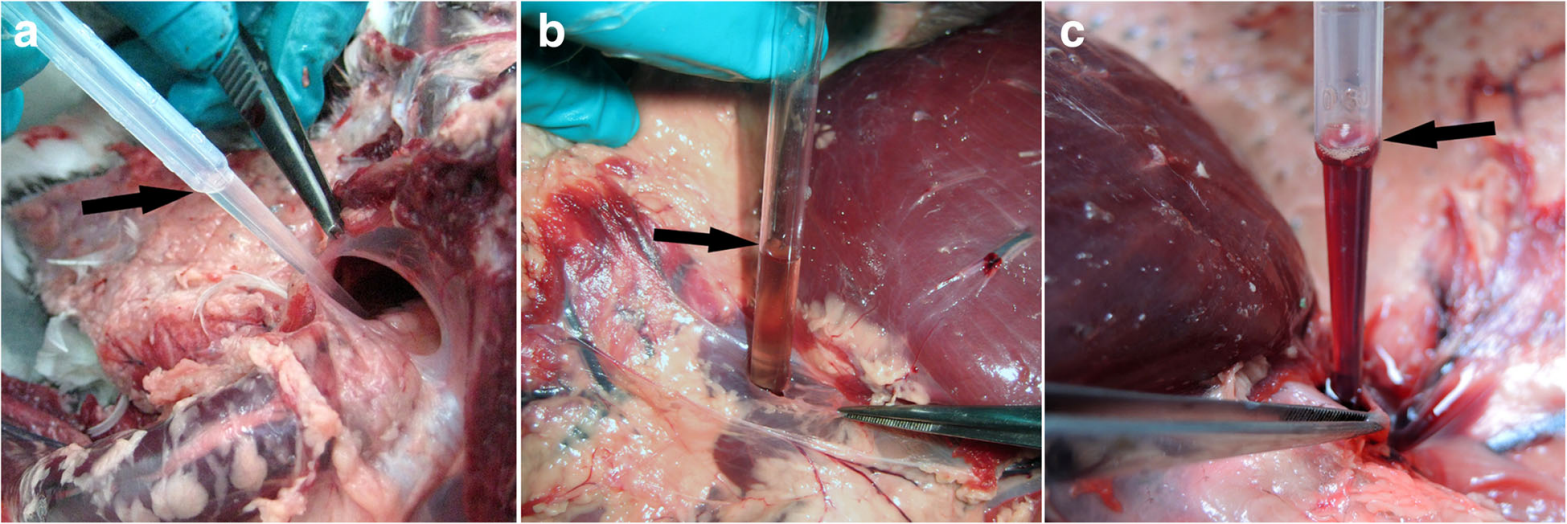
Variation in appearance of the fluid in the air sacs of drowned birds. These three GN divers died in the same incident and were submitted freshly dead. **a**: clear fluid is drawn from the clavicular air sac of a freshly dead bird. **b**: This bird had been held at 4^o^ C for 24 h. The fluid is blood tinged and is being aspirated from the axillary diverticulum of the left clavicular air sac. **c**: This bird had been stored at minus 20^o^ C for two months; the fluid is being drawn from the axillary diverticulum of the right clavicular air sac and is dark red


The skin over the ventro-lateral surface of the neck and body was reflected and in every case the jugular veins were observed to be greatly distended with dark blood. Care was taken not to puncture the exposed clavicular air sac and, with the exception of the divers, when slight fluctuating pressure was applied to the abdomen, fluid was observed moving in the distended air sac of most birds (Fig. 1). In divers, the clavicular air sac appeared to be bi-lobed but did not extend far enough forward in the interclavicular space to be properly visualised; however, applying pressure to the abdomen did cause distention of subcutaneous air sacs in the axillae, presumed to be extra-thoracic diverticula of the clavicular air sacs. In all species, when the carcase was opened, watery fluid was also frequently seen in the other air sacs, in particular the abdominal air sacs. An example of this can be viewed in an additional movie file [see Additional file 1]. In freshly dead or slightly autolysed specimens the fluid was normally watery, non-frothy and completely clear or slightly blood-tinged. In birds that had been refrigerated for 24 h it was usually slightly more blood-tinged whereas in birds that had been frozen it ranged from blood-tinged to dark red (Fig. 2). Watery fluid was recorded in the air sacs of over 86% (82/95) of cases; it may also have been present in some of the early incidents but not specifically noted. The volume of water varied from case to case but it was not considered practical to try to quantify it. A small amount of fluid, either clear or blood tinged, but not frothy, was occasionally seen in the lower trachea and bronchi; this was most marked in the GN divers.

Additional file 1: Gt N Diver B2337 Water in abdominal air sacs. In this movie, fluctuating pressure is being applied to the caudal abdominal wall of a GN Diver. The air sacs are distended with air and watery fluid can be seen moving within the bird’s right abdominal air sac. (MOV 14005 kb)


**Fig. 3:**
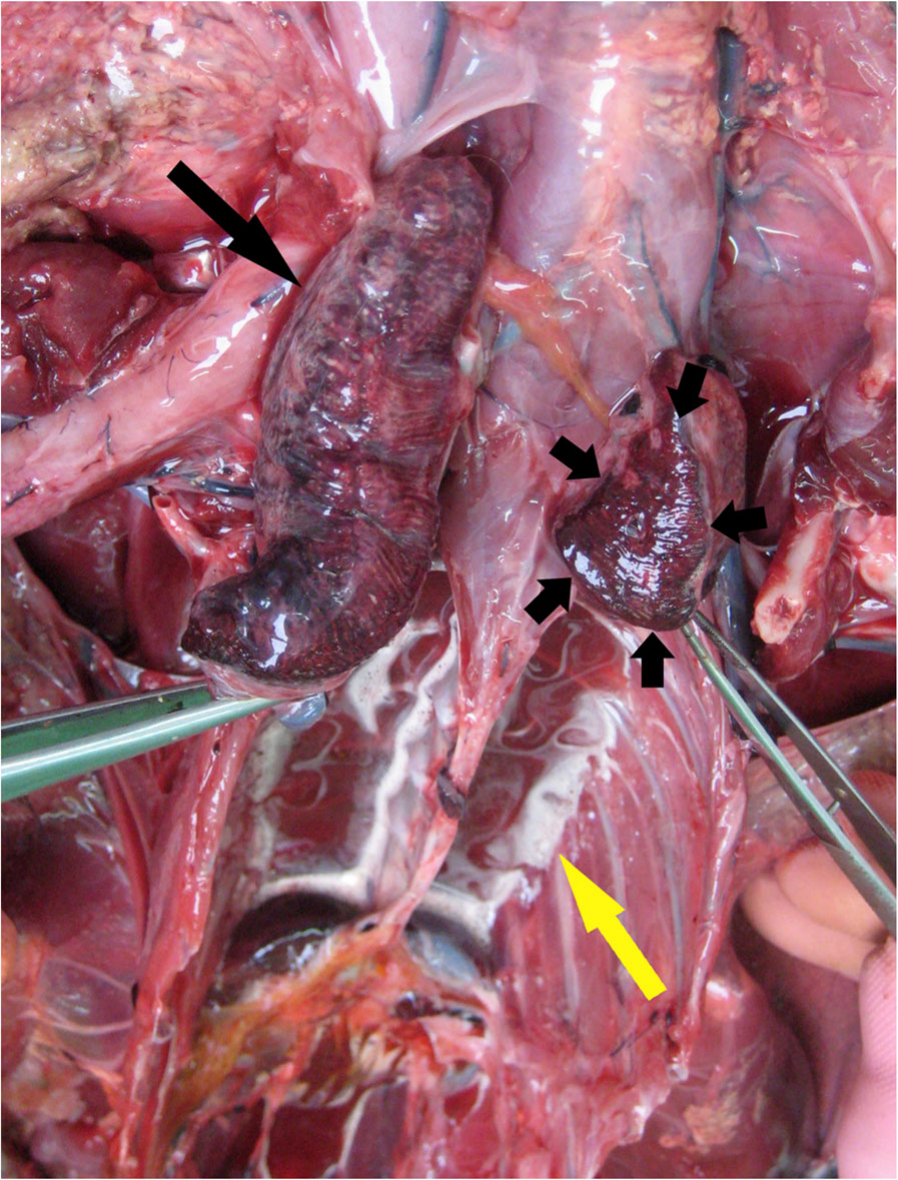
Thoracic cavity of a drowned shag showing swollen and intensely congested lungs. The lungs have been excised and moved cranially for examination. The right lung is shown intact (black arrow) but the left lung was bisected and the cranial half removed; the cut surface of the caudal half (small arrows) shows that the intense congestion extends evenly throughout the parenchyma. The white frothy fluid adjacent to the spine (yellow arrow) was released from the lungs when they were excised

**Fig. 4:**
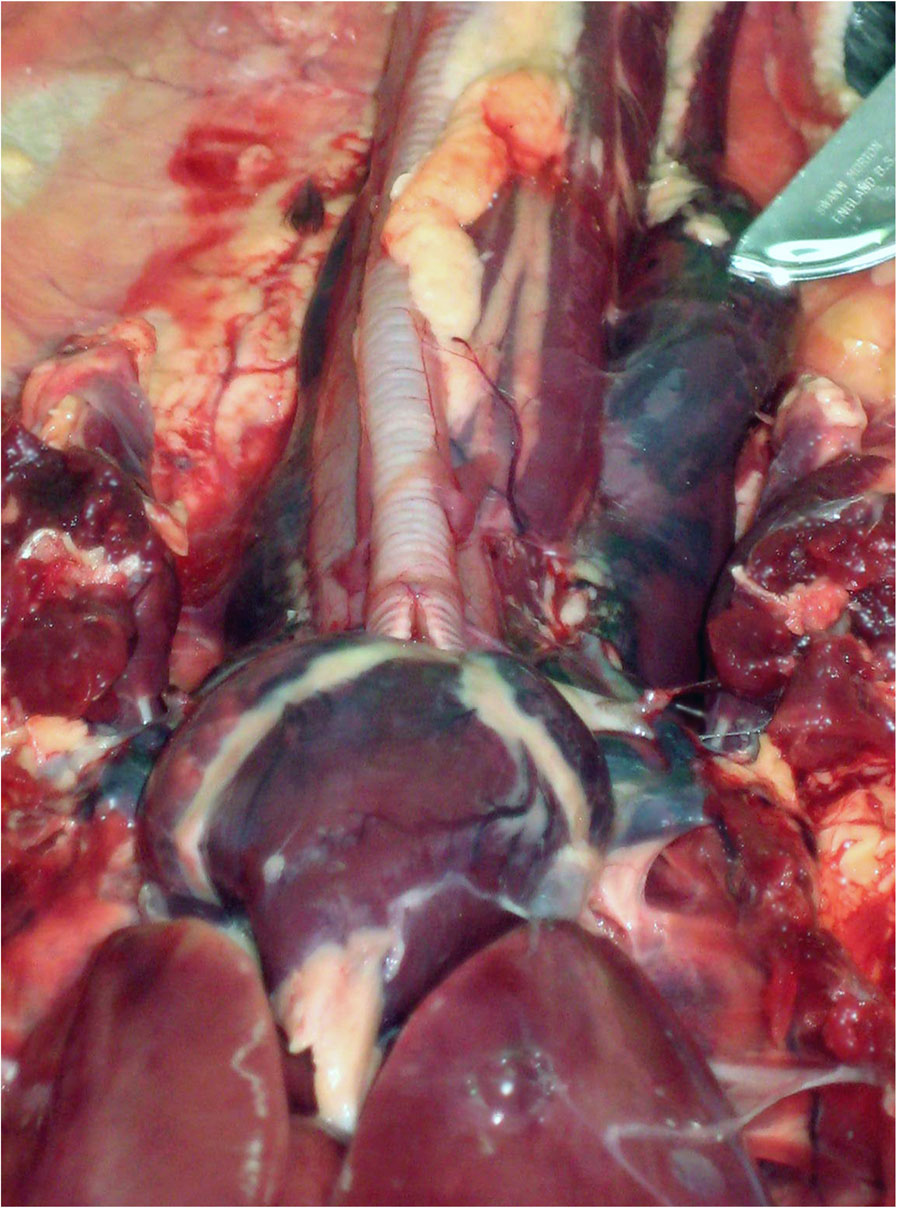
Ventral view of the organs at the base of the neck and cranial thorax of a drowned guillemot. The heart and cranial venae cavae are markedly distended with dark blood. The scalpel points to the distended left cranial vena cava. The right cranial vena cava is partially obscured

The lungs in all the confirmed drowned birds were swollen, intensely congested and oedematous and, when excised or sectioned, fluid exuded from cut surfaces. The fluid in freshly dead birds was usually white and frothy (Fig. 3). It was blood tinged in slightly autolysed ones and deeper red and less frothy in birds that had been frozen. In all cases, the cranial and caudal venae cavae and the chambers of the heart, especially the right atrium, were grossly distended with dark blood (Fig. 4).

**Fig. 5:**
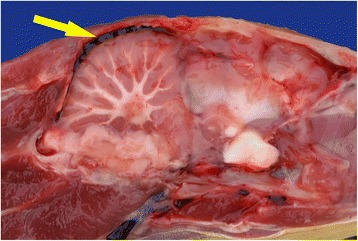
Sagittal section of the head of a drowned shag. The occipital sinus is greatly distended with dark blood (arrow). Reflection of the cranium showed widespread congestion of the caudal dural venous sinuses with dark blood

Congestion of the kidneys, and to a lesser degree the liver, was common. In the incident involving 26 shags, the heads of four shags, the two guillemots and the razorbill were cut in the mid sagittal plane and in each case the space between the dorsal surface of the cerebellum and the cranium, believed to be the occipital sinus, was distended with dark blood (Fig. 5). Removal of the cranium showed the other dural venous sinuses over the caudal brain to be markedly congested with blood.

Approximately half the birds in each of the confirmed drowning incidents had small fish, mostly Clupeid or Ammodytes-type, in their oesophagus, proventriculus and gizzard and these varied from being freshly ingested to partly digested. Otoliths were often present in otherwise empty gizzards. Large pale nematodes, identified as *Contracaecum rudolphi*, were frequently present in the proventriculus and gizzard of the shags, often in large numbers. No lesions were seen in the intestines. Overall, there were no notable differences between the species in the pathology due to drowning, although the distension of the venae cavae was more pronounced in the auks.

### Unconfirmed drowning

Six birds were examined from four unconfirmed drowning incidents. In the most significant of these 39 dead razorbills washed ashore at the fishing port of Mevagissey. Two were submitted for examination. Both birds had waterlogged feathers, were in a good nutritional state and had freshly ingested fish in the proventriculus. Their lungs were intensely congested but the possible presence of water in air sacs was not recorded. The other three unconfirmed incidents were of a minor nature involving just four birds, a guillemot, a razorbill and two shags. Both the shags were in a good nutritional state and one had freshly ingested fish in the proventriculus and gizzard. The lungs in both birds were intensely congested and produced white frothy fluid when excised. There was subcutaneous bruising around the base of the neck of one shag. The razorbill was in fair body condition and had a moderate quantity of part-digested sand eels (*Ammodytes* sp.) in the stomach. It had several large blood clots around the base of the neck and cranial thorax. Meaningful examination of the internal organs was not possible due to autolysis. The guillemot was in excellent body condition with congested lungs and watery fluid in the air sacs. As the birds in the unconfirmed drowned category had post-mortem lesions that were largely consistent with those in the confirmed drowning category, both groups are classed as drowned birds in the rest of this paper.

### PIB pollution

**Fig. 6:**
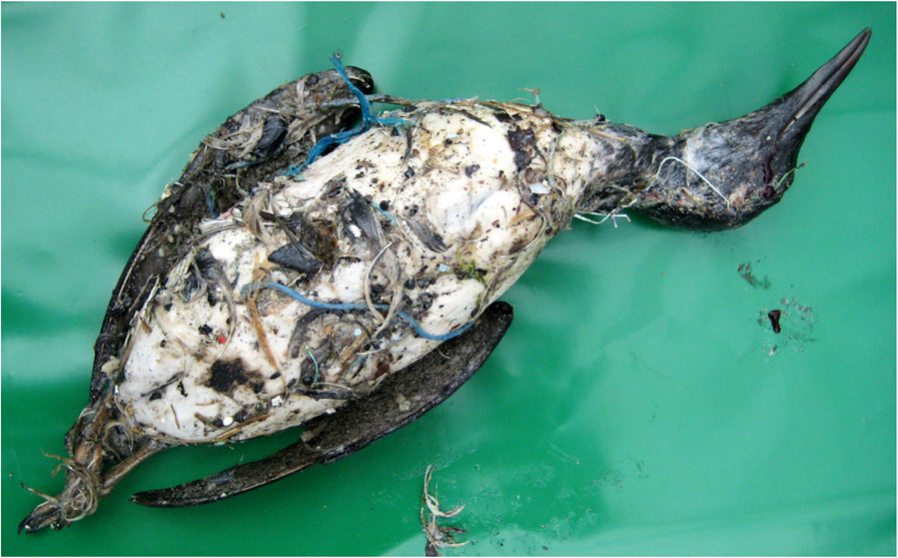
Ventral view of a guillemot contaminated by PIB. There is a thick layer of the gluey compound coating the whole body surface adherent to which are small stones and a variety of marine debris. The bird was in good muscular condition and had excellent subcutaneous fat deposits

Three frozen guillemots from a confirmed PIB spill were examined. The entire body surface of all three birds was coated in a thick layer of the whitish, glue-like, material; adhering to this were numerous stones, fragments of plastic and other marine debris (Fig. 6). The extreme stickiness of the coating meant that the birds’ limbs could not be extended or moved freely away from their body. The carcases showed mild autolysis, their subcutaneous fat deposits were good to moderate and muscular condition was good. In each case, the chambers of the heart and the cranial and caudal venae cavae were greatly distended with dark blood and the lungs were swollen and intensely congested. There was a moderate to large amount of dark blood-stained fluid throughout the body cavity but, due to changes caused by autolysis and freezing, it was not possible to say whether there may have been fluid in the air sacs ante-mortem. The livers appeared normal or slightly congested but the kidneys were markedly congested. Chronic granulomatous lesions up to 15 × 20 mm were present in the mucosa of the upper or mid in oesophagus of two birds; beneath the granulomatous layer there was ulcerated tissue in which was embedded unidentified small nematodes. One of the birds had four small sprat-like fish in the proventriculus and approximately 20 g of digested food in the gizzard but neither of the other birds had a significant amount of food in the upper alimentary tract. The post-mortem findings in the PIB-affected birds were largely consistent with those seen in confirmed cases of drowning and it was concluded that the ultimate cause of death was drowning, probably because the PIB had prevented the birds from moving their limbs. However, they could also have been affected to some degree by hypothermia and starvation.

### Oil pollution

**Fig. 7:**
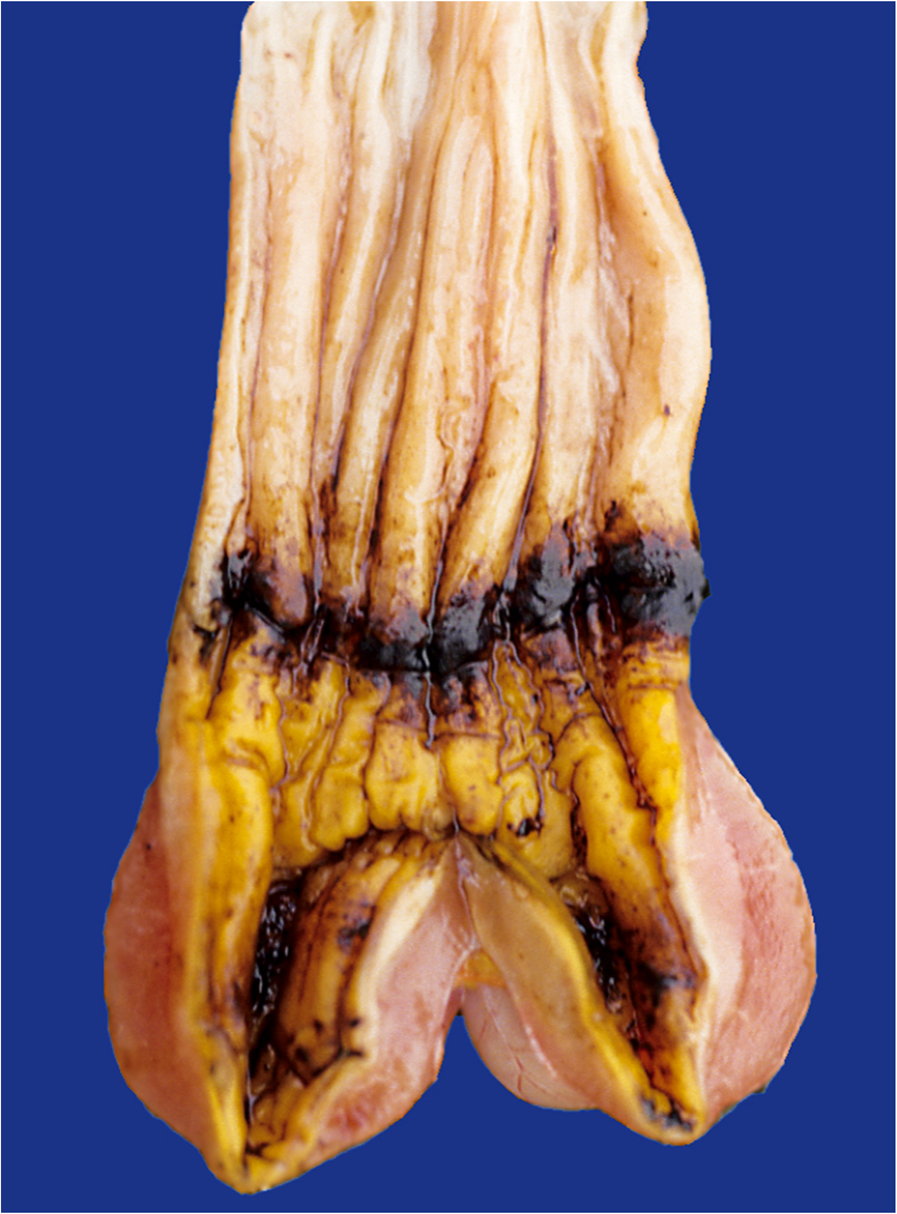
The opened proventriculus and gizzard of an oiled guillemot. The mucosa over the isthmus region is discoloured dark reddish brown and ulcerated. There was blackish, thick fluid in the lumen of the gizzard and this extended into the intestines

All but five of the 53 birds that died following exposure to oil were guillemots, the others being three razorbills and two shags. Most of the oiled birds showed a consistent pattern of lesions, irrespective of whether they had been examined immediately after being found stranded or after they had died or been euthanased in rehabilitation centres. There were no significant differences in the post-mortem findings between the species. Subcutaneous fat deposits were either minimal or absent and skeletal muscles were wasted. The heart and major blood vessels were unremarkable. In most cases the respiratory system appeared normal but two guillemots had lesions in the thoracic air sacs consistent with aspergillosis; both of these birds had been in extended hospital care. The livers of oiled birds were noticeably small and gall bladders were often distended with bile. There was normally no food in the upper alimentary tract but in some hospitalised birds there was a small amount fish in the proventriculus and gizzard. In almost half (20/45) of recorded cases, the mucosal surface of the isthmus, at the junction between the proventriculus and gizzard, showed changes that ranged from reddish brown discolouration (Fig. 7) to marked inflammation, haemorrhage and ulceration: nematode worms were sometimes observed in the submucosa beneath the lesion. In most instances the lesion was associated with blackish or reddish-black fluid that extended from the proventriculus and gizzard to the intestines. Severe inflammation and thickening of the small intestine was also common. There were no consistent lesions in other organs but the adrenal glands in some birds appeared enlarged.

### Unknown cause

Fourteen birds, 12 guillemots, one shag and one razorbill were submitted in six stranding incidents where the cause was not apparent. The post-mortem lesions in the guillemots were largely the same as those seen in the oiled birds. They were in poor muscular condition with no significant subcutaneous fat deposits and little of no food in their alimentary tracts. In one incident, which involved around 30 guillemots, three out of the four birds examined had caseous, necrotic ulcers in the oesophagus associated with fourth stage larvae of *Contracaecum* sp. Three guillemots in another incident had lesions of the isthmus consistent with those seen in many oiled birds. The shag had died in emaciated condition with yellowish jelly in the proventriculus and the razorbill had extensive respiratory lesions indicative of aspergillosis. However, this bird had been hospitalised and the lesions could have been of a secondary nature.

### Storm wreck

The single storm wreck incident involved six shags that were found dead together on a sheltered beach following a period of extremely stormy weather. Their plumage was clean with no evidence of oil. All six birds were in poor muscular condition with no subcutaneous fat. Several had subcutaneous masses of *Laminosioptes* sp. mites. Two had extensive haemorrhages around the upper neck and puncture wounds to the neck and chest consistent with attack by a mammalian predator. The alimentary tracts were empty save for a small amount of blackish bloody fluid and numerous nematodes identified as *C. rudolphi* in the gizzards. No lesions were seen in heart, kidneys or spleen but the livers appeared regressed. In five birds the respiratory tract appeared normal but one bird had extensive lung and air sac lesions consistent with aspergillosis.

### Examination of air sac fluid

Watery fluid taken from the air sacs of two razorbills that died in a confirmed drowning incident showed unlysed erythrocytes and unidentified, motile, plankton-type organisms. However, fluid samples taken from the air sacs of two guillemots and a razorbill that died in a second confirmed incident and of a guillemot and a shag in third confirmed incident all proved negative for marine organisms.

### Relationship between body and organ weights and cause of death

**Fig. 8:**
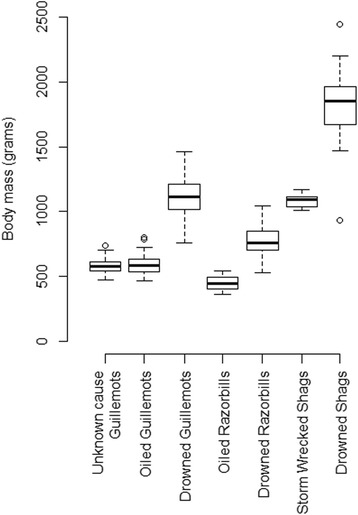
Variation of body mass in relation to cause of death. The body mass (Q1 and Q3, inter quartile range and median values) of birds that drowned was significantly greater than that of birds dying of other causes. Combinations of species and cause of death not plotted if there were less than three individuals

Most of the birds that died due to drowning had good subcutaneous fat deposits, well developed muscles and were heavier than birds that died of other causes (LM, F_1127_ = 565.7, *p* < 0.001). (Figure 8, see also Table 2). In all species males were heavier than females (LM, F _1127_ = 7.4, *p* = 0.008), with the sex-species interaction being non-significant (LM, F_2,124_ = 1.7, *p* = 0.196). Adults were heavier than immature birds in all species (LM, F_1127_ = 6.9, *p* = 0.01), with the species – age interaction being non-significant (LM, F _2122_ = 0.4, *p* = 0.656; Table 3). There were differences among the species in body mass (LM, F_2,127_ = 333.0, *p* < 0.001). Shags were heavier than guillemots (LM, F_1, 105_ = 391.7, p < 0.001), and razorbills (LM, F_1, 50_ = 600.7, p < 0.001), and guillemots were heavier than razorbills (LM, F_1, 96_ = 92.1, p < 0.001). Pectoralis muscle mass was only recorded only in drowned birds and, as this did not assist in comparison with other causes of death, the data are shown in Additional file 2 and Figure S1.

**Table 2:** The body mass, liver mass and pectoral muscle mass of the different species within the various categories of death

Species	Mean body mass g (Range, sd, n)			
	drowned	oiled	Unknown cause	Storm wreck
guillemots	1112.4 (760–1461, 157.3, 43)	592.5 (468–800, 73.6, 48)	586.2 (470–740, 76.2, 12)	NA
shags	1814.7 (930–2448, 272.9, 27)	NA	1046 (NA, NA, 1)	1086.3 (1010–1168, 56.3, 6)
razorbills	783.7 (530–1045, 125.2, 26)	448.3 (360–542, 91.1, 3)	455 (NA, NA, 1)	NA
	Mean liver mass g (Range, sd, n =)			
guillemots	50.6 (28.8–75, 10.3, 35)	22.8 (11.4–43, 9.0, 21)	24.3 (14–41.7, 10.0, 6)	NA
shags	72.4 (55.8–102, 10.5, 26)	NA	NA	30.6 (26.6–33.1, 2.6, 5)
razorbills	33.3 (20–51.4, 9.6, 10)	19.4 (NA, NA, 1)	NA	NA
	Mean pectoral mass g (Range, sd, n =)			
guillemots	156.2 (127.2–177, 17.1, 11)	NA	NA	NA
shags	180.8 (136.2–236.4, 22.2, 25)	NA	NA	NA
razorbills	95.1 (82.3–103, 9.13, 4)	NA	NA	NA

*NA* No data obtained

**Table 3:** The body mass by age class and sex of drowned and non-drowned guillemots and shags

	Mean body mass g. (Range, sd, n =)		
	Drowned Guillemots	Non-drowned Guillemots	Drowned Shags
Adult males	1148.3 (1002–1354, 118.9, 11)	613.6 (468–800, 96.2, 9)	1965.9 (1794–2448, 152, 14)
Adult females	1124.1 (794–1461, 167.0, 14)	577.2 (483–740, 68.14, 17)	1687.6 (1563–1798, 87.3, 7)
Immature males	1042.3 (851–1214, 121.1, 6)	599.5 (528–713, 53.3, 11)	1915.3 (1694–2200, 258.9, 3)
Immature females	1070.4 (810–1320, 233.5, 5)	558.8 (470–615, 54.1, 5)	1492.5 (1466–1519,37.5, 2)

**Fig. 9:**
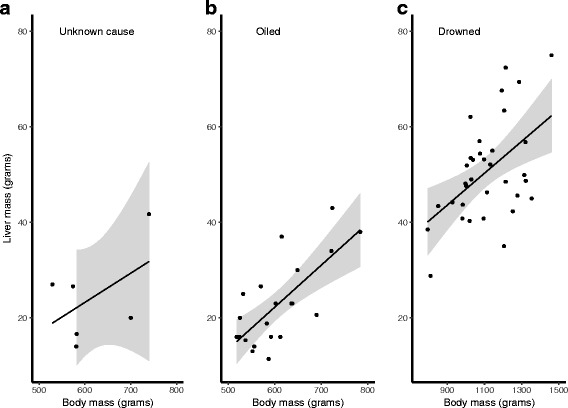
Body to liver mass relationship in guillemots dying of different causes. Plots show the significant, positive relationship between body and liver mass. Light grey areas indicate standard errors around the regression line. Birds that died of drowning had heavier livers than other birds for a given body mass, while the body mass – liver mass relationship did not differ between birds that died of drowning compared to other causes

**Fig. 10:**
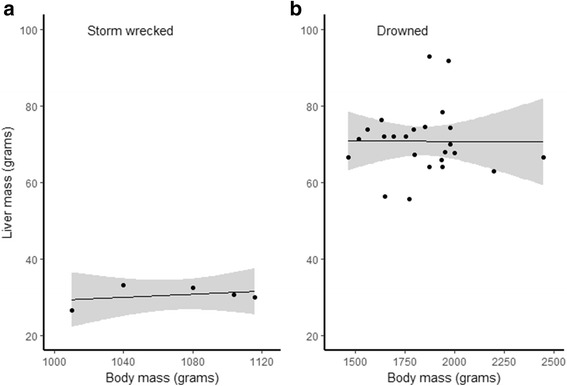
The plots show the relationship between liver and body mass in storm-wrecked shags compared with that in drowned shags

Where birds died in poor or emaciated condition a low body mass was not due solely to reduced or absent fat deposits and regressed skeletal muscles but to regressed internal organs such as the liver. It was noticeable when examining oiled or unknown cause guillemots, for example, that their livers often appeared small and weighing confirmed this, their mean mass being 22.8 g and 24.3 g respectively compared with 50.6 g in those that had drowned (Table 2). Liver mass was positively related to body mass (LM, F_1,98_ = 14.6, *p* < 0.001) and birds that died of drowning had heavier livers for a given body mass (LM, F_1,98_ = 18.3, *p* < 0.001) (Figs. 9 and 10). Liver mass was not influenced by age class (LM, F_1, 91_ = 0. 3, *p* = 0.590) or sex (LM, F_1, 97_ = 1.0, *p* = 0.325). Birds that died of drowning did not have a different relationship between body and liver mass to those who died of other causes (LM, F_1, 96_ = 0.7, *p* = 0.418). Controlling for body mass, there were species differences in liver masses (LM, F_2,98_ = 5.1, *p* = 0.007). Razorbills had smaller livers for a given body mass than guillemots (LM, F_1,69_ = 4.1, *p* = 0.047) and shags (LM, F_1,37_ = 9.8, *p* = 0.003), which were not different (LM, F_1,88_ = 0.5, *p* = 0.477).

All data used in statistical analysis is included in Additional file 3.

## Discussion

Much of the recorded pathology relating to drowning and other causes of mass mortality of seabirds is derived from studies where specimens were frozen prior to post-mortem examination and where, in many cases, the birds were affected by a variable degree of autolysis [7–11]. A consequence of this is that some lesions, especially those affecting the respiratory system, become obscured by artefactual changes. Descriptions of lesions in birds known to have drowned and where at least some of the carcases were examined in fresh, unfrozen, condition are uncommon. In this study, the majority of the 95 birds known to have drowned were examined in fresh condition. The changes seen in the respiratory system showed a high level of uniformity and were considered to be entirely consistent with the inhalation of seawater.

When a bird inhales under normal circumstances, fresh air passes along the primary bronchi and enters, via openings known as ostia, directly in to the caudal air sacs (caudal thoracic and abdominal sacs); air from some secondary bronchi and parabronchi also passes, via the ostia, in to the caudal air sacs. On exhalation, most of the air (more than 88%) in the caudal air sacs passes, via secondary bronchi and their parabronchi, in to the lung parenchyma; only a small amount (less than 12%) escapes via the primary bronchi and trachea [12]. During the next phase of inhalation, the air in the parabronchi and the parenchyma, having undergone gaseous exchange, is drawn via secondary bronchi, in to the cranial air sacs (cervical, clavicular, and cranial thoracic air sacs). Fresh air is prevented from entering the cranial air sacs directly from the primary or secondary bronchi due to a system of valves [12, 13]. Assuming that inhaled seawater behaves in much the same way as air, most of the water found in the cranial air sacs of the known or suspected drowned birds in the present study must have passed through the pulmonary parenchyma after being expelled from the caudal air sacs.

It might be argued that if a bird had died at sea from causes other than drowning water could have entered the respiratory system, and in particular the air sacs, after it had died. The evidence does not support this. Many of the oiled and unknown cause birds in this study appeared to have died at sea yet none had anything more than a small amount of water beyond the proximal trachea. None had water in their air sacs. Even if water was able to passively enter the air sacs post-mortem, this would not result in the pathological changes seen in the lungs and cardiovascular system of drowned birds. As part of the standard post-mortem protocol followed in this study [5], all the birds were thoroughly washed in warm water and detergent and this included immersion of their head; despite this, watery fluid was only seen in the air sacs of the drowned or suspected downed birds.

The presence of a large amount of froth or frothy fluid around the nose, mouth and/or in the airways is a well-recognised feature of humans and other mammals that have drowned in sea water; however, it is not considered to be pathognomonic for drowning [14, 15]. A small amount of watery, clear, slightly frothy fluid was occasionally present in the lower trachea and bronchi of the drowned birds but, in contrast to what often happens in drowned mammals, it never extended up the trachea or into the mouth and nares. In mammals, when sea water enters airways it destroys the surfactant coating and, because it is hypertonic to plasma, water is drawn, together proteinaceous fluid, from the lung parenchyma in to the alveoli; this results in haemoconcentration and hypovolaemia [14]. In birds, surfactant occurs mostly lining the walls of the parabronchial atria and the air capillaries. It is presumed that it is this, admixed with seawater and protein-rich fluid released from the parenchyma, that forms the white frothy fluid seen when the lungs of drowned birds are excised or bisected. The release of this fluid from the lungs, together with gross pulmonary congestion and oedema, was a constant feature in the drowned birds. However, in drowned birds affected by autolysis, and especially those that had been frozen prior to examination, the normally white fluid ranged from blood tinged to deep red and tended to be less frothy.

In all the drowned birds, including the PIB affected guillemots but not those birds dying of other causes, the major veins and the chambers of heart, especially those on the right side, were markedly distended with dark blood. It is considered that these changes could be indicative of acute circulatory failure and hypoxaemia, possibly as a result of back pressure on the pulmonary arteries due to a combination of extreme pulmonary congestion, oedema and haemoconcentration. The marked congestion of the occipital and other dural venous sinuses observed in drowned birds is, in the authors’ experience, unusual and is not normally seen in birds dying of other causes. However, as the dural venous sinuses drain via the caudal cephalic veins directly in to the jugular veins [16], the congestion of the sinuses is likely to be a result of drowning.

In the oiled birds, the predominant post-mortem lesions of emaciation, blackish fluid in empty or near-empty alimentary tracts and enteritis were consistent with those recorded in birds known to have been exposed to oil [7, 17–19]. However, the same or very similar lesions were also frequently seen in the non-oiled, unknown cause, birds in the present study and they cannot be considered specific for oil exposure. The nature and origin of the blackish fluid is not clear. In a study of oiled rhinoceros auklets (*Cerorhinca monocerata*) blackish fluid in the gut was shown not to be ingested oil [7]. Similarly, analysis of black fluid in the intestines of oiled guillemots and razorbills proved negative for hydrocarbons but positive for occult blood [19]. In ducks experimentally dosed with oil, the dark gut fluid also proved positive for occult blood: no discrete focal lesions were present and the authors concluded that blood had entered the gut by diapedesis [18]. In the present study it is likely that the blackish fluid in the gut was, at least in part, due occult blood as a result of haemorrhage from an inflamed or ulcerated isthmus or, where there were no visible haemorrhagic lesions, as a result of diapedesis. Blackish-red fluid is commonly present in the gut of birds of prey dying of starvation where it is believed to be stress-related [20]. Stress was thought to be responsible for histological lesions in the adrenal glands of birds experimentally dosed with crude oil [21]. It is suggested that stress may also contribute to the formation of blackish fluid in the gut of oiled or debilitated sea birds.

Birds exposed to oil, whether by contamination of plumage or by ingestion, suffer severe weight loss [7, 22, 23], in part due to hypothermia, impaired mobility, starvation and stress but also due to the direct toxic effect of oil on internal organs [21, 22, 24]. The oiled birds in this study were almost all in debilitated condition with nil or negligible amounts of subcutaneous fat, wasted muscles and regressed internal organs. Both their mean body mass and liver mass was significantly lower than that in drowned birds which, in almost every case, had good subcutaneous fat, well-developed muscles and normal looking internal organs. However, the condition of birds that had died due to adverse weather or that had become stranded due to unknown cause was not significantly different from that of the oiled birds. The mean body mass of oiled and unknown cause guillemots (592.5 g and 586.2 g respectively) was approximately half that of the drowned birds (1111.6 g). The fact that the emaciated, non-drowned birds had lighter livers than expected for a given body weight suggests that liver atrophy started to occur at an early stage of illness. This has been recognised in debilitated waterfowl elsewhere [25]. Although the body mass of the drowned guillemots would have been influenced to some degree by the fact that their plumage was often wet, and possibly by the presence of some residual ingesta, the mean was very similar to that of healthy adults (1107 g) and immatures (877 g) wintering in Scotland [26]. It is considered unlikely that the volume of water in the air sacs of drowned birds relative to their body mass would have significantly affected these results.

The post-mortem lesions in the oiled birds and the unknown cause birds in the present study were similar to those reported in two studies of guillemots that stranded dead in Belgium [8, 9]. In both the Belgian studies, weight loss, cachexia and haemorrhagic gastroenteritis were the most common findings but there was no clear correlation between these lesions and the presence of oil, either on the plumage or in the gut [8, 9]. In one study [8] the mean body mass of the cachectic birds was 708 g compared with 781 g in birds considered non-cachectic. Although oiling was considered to be the main cause of death, no cause was determined in the 45% of birds that were not oiled, many of which had haemorrhagic enteropathy lesions [8]. In the other Belgian study [9] birds showing cachexia and haemorrhagic gastroenteritis had lower a body mass than non-lesioned birds; however, although the authors classed the non-lesioned birds as ‘normal’, they acknowledged that their mean body mass was around 25% less than the mean of 1031 g seen in healthy guillemots shot in Scotland [27]. The non-oiled, cachectic birds in both these studies would appear similar to the unknown cause birds in the present study. The authors of both the Belgian studies suggest that the emaciated state of the non-oiled birds could have been due to a variety of factors including food deprivation, adverse weather and mild chronic exposure to oil [8, 9]; however both studies were carried out during the winter months when the body mass of guillemots should have be at their maximum [26, 27]. Another possible explanation for some of the unknown cause deaths in both the present study and those in Belgium is that they were originally oiled birds which had been treated in a rehabilitation centre but had then died post release. Various organisations regularly treat oiled seabirds in Cornwall but is well recognised that the post release survival rate in auks is poor [28].

The six shags diagnosed as storm wrecked were found in the sheltered Helford creek during the so-called ‘Burns Day Storm’ in January 1990. The storm affected most of north-western Europe and is one of the strongest European windstorms on record. Storm wrecked birds are typically in poor emaciated condition and have little or no fat deposits, presumably because of difficulty in feeding in turbulent, often turbid, water and also prolonged physical exertion [29]. Two of the Helford shags had bite wounds and subcutaneous haemorrhage to the upper neck and pectoral muscles. It seems likely that in their weakened state they had been killed by a predator, possibly an American mink (*Mustela vison*). Although storm wrecks have long been recognised as natural events [30], incidents such as the one in 1969 which killed around 17,000 guillemots in the Irish Sea, led to research which also implicated the possible role of pollutants, in particular polychlorinated biphenyls [31, 32]. Subsequent studies have also shown that storm-wrecked birds often have high tissue levels of heavy metals, in particular mercury, and as a result may be less able to cope with adverse weather [33]. However, despite much research, the role, if any, played by pollutants in seabird wrecks is not clear.

Exposure of birds to PIB contrasts with that due to oil in that PIB is relatively nontoxic; it is a synthetic polymer used to make many products including sealants, chewing gum, cosmetics and adhesive tape. In its raw state is hydrophobic but when mixed with sea water it forms a viscous glue-like layer that floats just beneath the surface. As with oil, birds become exposed to PIB when it is discharged from ships’ tanks. The body condition of the PIB-contaminated birds in this study was good and this is consistent with the observation in a previous PIB incident, in the Netherlands in 2010 where the birds died in “*excellent physical condition (large deposits of fat, muscular breast, good organ condition, full stomachs), indicating rapid death*” [34]. The rapidity of death is almost certainly a result of the immobilising effect of a heavy coating of PIB.

## Conclusion

The lesions in drowned birds in this study were mostly similar to those reported in three earlier studies [10, 34, 35] but were probably more easily recognised as the majority were examined in fresh condition whereas the birds in the previous studies were mostly frozen. One notable difference was that skeletal fractures and haemorrhages, which were common features of drowned birds in North America and New Zealand [35, 36], were rarely seen in this study. In human and veterinary mammalian pathology it is generally accepted that there are no post-mortem lesions that are pathognomonic for the diagnosis of drowning [14, 37]. However, it is perhaps pertinent to question whether this should be applied to avian pathology, especially when birds drown in seawater. In the author’s experience the presence of a significant amount of clear watery fluid in the air sacs is only seen in cases of drowning. Whilst other causes of mortality such as asphyxia due smothering, polytetrafluorethylene (PTFE) toxicity, chemical irritation or acute infections such as yersiniosis may result in pulmonary lesions that can resemble, to some degree, those of drowning, none are associated with a significant amount of clear watery fluid free in the air sacs. The authors accept, however, that where carcases are autolysed or have been frozen, or both, it may be difficult or impossible to distinguish fluid arising from inhalation of water from that arising as a result of inflammatory or toxic lesions, or as artefacts caused by autolysis, freezing and thawing. Nevertheless, in freshly dead birds, the combination of watery fluid in air sacs, lungs that are extremely congested, oedematous and releasing white frothy fluid, and marked distension of the heart and major veins, should be taken as strong indication of death from drowning. If, in addition, the bird is one of a batch that died in good physical condition with freshly ingested fish in their upper alimentary tract, it is the authors’ opinion that a diagnosis of drowning may be made with a degree of confidence. The pathologist might wish to try to confirm the diagnosis by demonstrating plankton, diatoms or other contaminants in the respiratory system. However, water taken from the air sacs of several confirmed drowning cases in the present study gave conflicting results. These results tend to support the widely held view among forensic pathologists that diatom testing to confirm death by drowning is unreliable [14, 15, 38, 39].

Public anger and distress following major wildlife mortality incidents, together with concern over declining fish stocks, undoubtedly act as drivers for increased protection of marine species. Following a series of bycatch incidents at St Ives, Cornwall, a byelaw was made in 1999 which allows the imposition of a temporary - but significant - ban on all fishing in the event of more than a specified number of birds being caught in nets [40]. The discharge of PIB along the coast of Cornwall and Devon in 2013 caused the death of at least 4200 birds. Previous incidents had occurred elsewhere in Europe in 1994, 1998 and 2010 but no action against the polluters was possible as it was legal at that time for ships to discharge at sea. The 2013 incident led to concerted lobbying of the UK Government by a range of conservation organisations and public groups and resulted in the International Maritime Organisation of the UN imposing an international ban in 2014 on the discharge of high viscosity PIB [41]. These two examples demonstrate what can happen when there is a very public mortality incident and where the cause is proven. By contrast, chronic low level mortality, irrespective of the cause, tends to attract little media attention and as a result no action is taken.

Seabirds face ever increasing threats from human activities and in the European Union (EU) there have been recent moves aimed at providing more protection. One of the requirements of the EU Birds Directive (2009/147/EC) is that Member States designate as Special Protection Areas (SPAs) the most suitable territories for rare or vulnerable species (as listed in Annex I of the Directive, Article 4.1) and for all regularly occurring migratory species (Article 4.2) [42]. In a second piece of legislation aimed at protecting marine wildlife, the European Commission stated its intention to impose a complete ban on the use of drift nets in EU waters in 2015 [43]. However, neither piece of legislation imposes any requirement on Member States to systematically look for and examine stranded seabirds. Fishermen are not required to report bycatch and, without data on the frequency or causes of mortality and the species involved, the regulatory authorities are unlikely to have the evidence needed to enforce controls on human activities that are harmful to birds. It is apparent that if the legislation is to be properly implemented there needs to be systematic collection and meaningful examination of stranded seabirds. By providing a clear description of the lesions that characterise death by drowning it is hoped this report will assist anyone performing post-mortem examinations, whether veterinarians, zoologists or ecologists, to determine whether or not the birds have drowned.

## Additional files

Additional file 2: Relationship of pectoral muscle mass to body mass in drowned birds by species. These data allow comparison of pectoral muscle mass to body mass in apparently healthy birds. **Figure S1** Correlation between body mass and pectoral mass. Description: Correlation between body mass and pectoral mass of guillemots, razorbills and shags that had drowned. (DOCX 883 kb)

Additional file 3: This file shows all the raw data used in statistical analyses in this study. (XLSX 20 kb)
