# Supplementary material for: A description of the gross pathology of drowning and other causes of mortality in seabirds
Source: BMC Vet Res. 2017 Oct 12;13:302. doi: 10.1186/s12917-017-1214-1 (PMC5639757; doi:10.1186/s12917-017-1214-1)
Supplement: Supplementary file 2 — Relationship of pectoral muscle mass to body mass in drowned birds by species. These data allow comparison of pectoral muscle mass to body mass in apparently healthy birds. Figure S1 Correlation between body mass and pectoral mass. Description: Correlation between body mass and pectoral mass of guillemots, razorbills and shags that had drowned. (DOCX 883 kb) [file 12917_2017_1214_MOESM2_ESM.docx]

Additional file 2

Pectoral muscle mass was only recorded in drowned birds and was positively related to body mass (LM, F_1, 35_ = 54.2, p < 0.001) (Figure 9). It was not influenced by age (LM, F_1, 34_ = 0.6, p = 0.455), but males had heavier pectoral muscles than females (LM, F_1, 35_ = 5.5, p = 0.025). Controlling for body mass, there were species differences in pectoralis masses (LM, F_2,35_ = 35.0, p < 0.001.). Controlling for body mass, age and sex, guillemots had heavier pectoral muscles than shags (LM, F_1, 32_ = 17.0, p < 0.001) and razorbills (LM, F_1, 11_ = 38.8, p < 0.001), which were not different (LM, F_1, 25_ = 0.4, p = 0.558) (Figure 1).


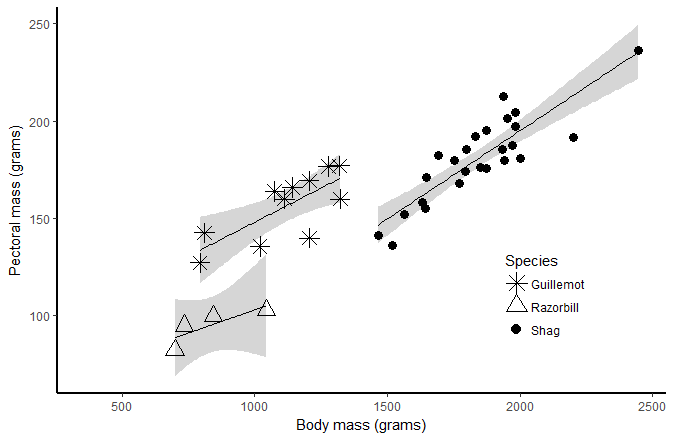


**Figure 1. Correlation between body mass and pectoral mass**

Body mass in all species was closely correlated with pectoral muscle mass. Grey areas indicate standard errors around the estimated regression line. Shags are indicated with solid circles, guillemots with 8-pointed crosses and razorbills with triangles. Relative to their body mass, guillemots had heavier pectoral muscles than shags and razorbills, which were not different.
